# Influence of Meat Spoilage Microbiota Initial Load on the Growth and Survival of Three Pathogens on a Naturally Fermented Sausage

**DOI:** 10.3390/foods9050676

**Published:** 2020-05-25

**Authors:** Luis Patarata, Margarida Novais, Maria João Fraqueza, José António Silva

**Affiliations:** 1 CECAV, Centro de Ciência Animal e Veterinária, 5001-801 Vila Real, Portugal; jasilva@utad.pt; 2 School of Agrarian and Veterinary Sciences, Universidade de Trás-os-Montes e Alto Douro, 5001-801 Vila Real, Portugal; luiscpatarata@gmail.com; 3 CIISA, Centro de Investigação Interdisciplinar em Sanidade Animal, Faculdade de Medicina Veterinária, Universidade de Lisboa, Avenida da Universidade Técnica, 1300-477 Lisboa, Portugal; mjoaofraqueza@fmv.ulisboa.pt

**Keywords:** naturally fermented sausage, spoilage microbiota, starter culture, lactic acid bacteria, wine, nitrite, foodborne pathogens, *Salmonella*, *Staphylococcus aureus*, *Listeria monocytogenes*

## Abstract

Meat products are potential vehicles for transmitting foodborne pathogens like *Salmonella*, *S. aureus*, and *L. monocytogenes*. We aimed to evaluate (1) the effect of the meat’s initial natural microbiota on *Salmonella*, *S. aureus*, and *L. monocytogenes* growth and survival in a batter to prepare a naturally fermented sausage, made with and without curing salts and wine (2) the effect of a lactic acid bacteria (LAB) starter culture and wine on the survival of the three pathogens during the manufacturing of a naturally fermented sausage made with meat with a low initial microbial load. The results revealed that the reduced contamination that is currently expected in raw meat is favorable for the multiplication of pathogens due to reduced competition. The inhibitory effect of nitrite and nitrate on *Salmonella*, *S. aureus*, and *L. monocytogenes* was confirmed, particularly when competition in meat was low. In any attempt to reduce or eliminate nitrite from naturally fermented sausages, the use of LAB starters should be considered to ensure an unfavorable competition environment for pathogens. In the experiment with naturally fermented sausage, *chouriço*, the reduction in a_w_ strongly inhibited the challenged pathogens, particularly when a LAB starter culture and wine were used.

## 1. Introduction

European and North American foodborne surveillance data point out meat and meat products as food vehicles that are frequently associated with foodborne disease outbreaks [1,2]. *Salmonella* is one of the pathogens most frequently found in outbreaks resulting from fermented sausage consumption. Due to the severity of the disease, widespread distribution in processing plants, and to the theoretical adequacy of the fermented sausage ecosystem to support its growth, *Listeria monocytogenes* must be considered as a potential hazard in the manufacturing of these products. *Staphylococcus aureus* can grow in foods with a reduced water activity (a_w_). Thus, it should be prevented when preparing fermented sausages [3,4,5].

The preventive hygienic measures implemented at slaughterhouses aim to achieve a low initial contamination and control the safety of meat. However, any pathogen presence during processing should be considered. When the initial microbial contamination on meat is low and no starter cultures are added, pathogens face less competition, increasing the odds of their fast multiplication [6,7]. The fermentation carried out by the natural microbiota of meat and by the so-called house microflora, or by the addition of starter cultures, combined with the reduction in water activity, are the main hurdles to several pathogens [8,9]. In the meat processing industry, it is common to use nitrite, alone or in combination with nitrate, to control *Clostridium botulinum* and due to their effect on color stabilization [10]. These chemical preservatives also have a reputed inhibitory effect on other foodborne pathogens with importance in the meat product industry, namely *Salmonella*, *L. monocytogenes*, and *S. aureus* [11,12]. The association between colon cancer and processed meats is mediated among other aspects by the eventual formation of N-nitroso-compounds in meat products prepared with nitrite [13]. Nowadays, the consumer demand for additive-free meat products is growing [14,15]. Potential health problems and consumer behavior have led to a trend towards eliminating or decreasing the level of nitrite in the meat industry. Nonetheless, despite the apparently favorable context to reduce or eliminate nitrite, the meat industry is still very reluctant to take this step. The fear of foodborne outbreaks and the expectation that consumers might appreciate products less due to unsatisfactory color are believed to be the main hurdles [16]. To reduce nitrite in dry sausages, it is important to understand how pathogens will be controlled to validate their safety. The safety of dry sausages relies on hurdle technology, a combination of several preservation techniques. In addition to the reduced a_w_ and the activity of lactic acid bacteria (LAB), other aspects can exert important hurdle effects on pathogen growth or survival, namely reduced temperature during processing and low pH, the use of preservatives, smoking, the eventual antimicrobial effect of spices and herbs, and wine [17]. In wine-producing regions, it is common to season dry sausages with wine [18,19,20,21,22]. Wine is claimed to have antimicrobial potential in marinated meat and meat products [19,23]. The antimicrobial effect of wine in dry-fermented sausage safety might be associated with its low pH and the presence of ethanol and phenolic compounds [24]. The survival and growth of pathogens is modulated by the ensemble of factors present in food. In the present work, we aimed to evaluate (1) the effect of the initial meat natural microbiota load on *Salmonella*, *S. aureus*, and *L*. *monocytogenes* growth and survival in a batter to prepare a naturally fermented pork sausage, made with and without curing salts and wine, and secondly, (2) the effect of a LAB starter culture and wine on the survival of the three pathogens during the manufacturing of a naturally fermented pork sausage made with meat with a low initial microbial load.

## 2. Materials and Methods

### 2.1. Experimental Design

**Figure 1:**
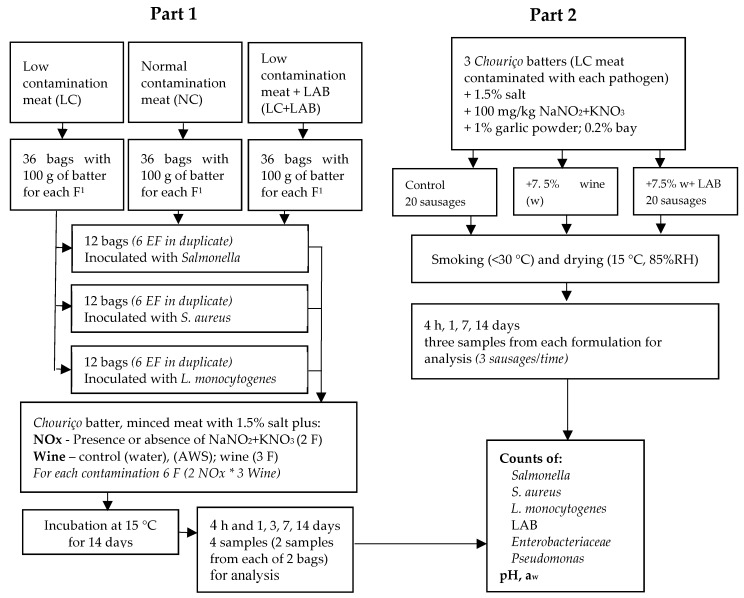
Experimental design. ^1^EF Experimental Formulation.

The experiment was composed of two parts. In the first part of the study, a batter similar to that used for naturally fermented pork sausage manufacturing was used to study the effect of the initial contamination of meat, nitrite, and nitrate and wine on the behavior of *Salmonella*, *S. aureus*, and *L. monocytogenes*. In the second part of the study, the effects of wine and wine plus a LAB starter culture on the behavior of the three pathogens in *chouriço* were studied. The design of the experiment is presented in Figure 1.

### 2.2. Bacterial Strains and Preparation of Inoculum

For each tested pathogen, a mixture of three strains was used (Table 1), one strain from a culture collection and two wild-type strains isolated from meat products or their production environment. For inoculation, 24 h single cultures in 30 mL of Brain Heart Infusion (BHI, Biokar, Allonne, France) were harvested by centrifugation, washed twice, and suspended in NaCl 0.85%. Mixtures of the three strains were prepared and diluted to achieve a level of inoculation of ca. 2.5 log cfu/g, as recommended for microbiological challenge testing [25,26]. *Lactobacillus sakei* (Lch45), which was previously tested for its antagonistic activity [27], was prepared similarly to the pathogens, but using De Man, Rogosa, and Sharpe (MRS) broth. The level of inoculation was adjusted to 6 log cfu/g.

The concentration of microorganisms was estimated by the turbidity of the suspension. It was adjusted to a 0.5 McFarland standard (Biomerrieux, Marcy-l’Etoile, France) and adequate decimal dilutions were made in NaCl 0.85% to achieve the required inoculation level. The number of microorganisms in the inoculation suspension was confirmed by seeding the pathogens in BHI agar: Inoculum of *L sakei* was serial diluted in NaCl 0.85% and seeded in solid MRS.

The concentration of microorganisms was estimated and confirmed as described for pathogens.

### 2.3. Part 1. Experiments with a Chouriço Batter

All experiments were prepared using meat and fat from pork belly. Pork bellies were purchased in a local retailer on the day of arriving from the abattoir. To prepare meat with low contamination (LC), pork bellies were immersed in ethanol; the surface of the meat was burned with a blowtorch, and the burned surfaces were excised under aseptic conditions. Only non-heated meat was used for further experiments. Normal contaminated meat (NC) was used as it was purchased. To assess the initial microbiota of meat, *Enterobacteriaceae*, *Pseudomonas*, and LAB were enumerated. The reductions obtained through the treatment were between 1 and 2 log cfu/g (see Section 3.1). Then, 12 kg of meat and fat from the pork bellies (4 kg of each contamination level) were ground (15 mm) (Mainca, Barcelona, Spain) and, for each initial microbiota contamination level (LC, NC, LC + BAL), distributed into 36 polyethylene bags containing 100 g. Twelve bags containing meat at each initial contamination level were inoculated with *Salmonella* strains, 12 bags were inoculated with *S. aureus* strains, and 12 bags were inoculated with *L. monocytogenes* strains. Inoculated meat was homogenized with a stomacher (3 min). For low contaminated meat with *L. sakei* (LC + LAB), the starter culture was added after inoculation with pathogens.

To guarantee the distribution of the tested ingredients, they were added individually to each bag. Sodium nitrite and potassium nitrate (hereafter referred to as NOx) were added to achieve a level of 150 mg/kg batter. To test the effect of wine (W), 7.5% red wine was used (pH 3.8, 12.5% ethanol) and a solution to imitate wine, thereafter called artificial wine solution (AWS), was prepared with ethanol (12.5%), tartaric acid (0.22%), lactic acid (0.20%), malic acid (0.12%), succinic acid (0.12%), and acetic acid (0.05%) [28]. Artificial wine solution was used to evaluate if the putative inhibitory effect of wine was associated with the ethanol and the combination of organic acids that are usually present in wine. Inoculated meat was seasoned with 1.5% salt. Other ingredients were added according to the experimental design. Two units of 100 g were prepared for each experimental condition. After removing the air, the bag was closed with a clip. The batters were incubated at 15 °C, which is a mean temperature usually used by several producers to cure/dry sausages [23]. Two samples of 5 g were withdrawn from each bag after 4 h and 1, 3, 7, and 14 days and prepared for analysis.

### 2.4. Part 2. Experiment with a Naturally Fermented Sausage—Chouriço

The naturally fermented sausage was prepared as described for the batter experiment. Meat inoculated with each pathogen was distributed in three batches. All samples were seasoned with 1.5% salt, 1% dried garlic, and 0.2% powdered bay leaves. Curing salts (5% NaNO_2_ and 5% KNO_3_) were added at 0.20% to achieve a level of 100 mg/kg in the sausage batter. Three formulations were prepared: Control, with no further ingredients; Wine, with 7.5% red wine; and Wine + LAB, with 7.5% red wine and 6 log cfu/g *L. sakei*. After mixing, the batter was filled into a thin natural pork casing and tied in a horseshoe shape (each sausage weighed ca. 200 g). The smoking occurred in a chamber (Begarat, Thermaxs 100EC, Berlin, Germany) with an electric resistor applied to fireless burn beech wood scraps for 3 h at a temperature between 25 to 30 °C. The drying was done in a climatic chamber at 15 °C, 85% RH (Aralab Fitoclima, Rio de Mouro, Portugal) until day 14.

Three sausages were collected for analysis at each experimental time 4 h after the batter’s preparation, after smoking (day 1), and after 7 and 14 days of drying.

### 2.5. Bacterial Enumeration

As the inoculation levels were low (near 2 log cfu/g) and close to the counting methods’ detection limit, an initial dilution of 1:5 was prepared in 0.85% NaCl. When low counts were expected, an inoculation of 0.5 mL of the first dilution was spread on two Petri dishes (0.25 mL each) and slightly dried in the laminar flow for 5 min to avoid biofilm formation during incubation. When high counts were expected, appropriate serial dilutions were used and the standard 0.1 mL inoculation was made. Compass *Salmonella*, Baird Parker with Rabbit Plasma Fibrinogen (RPF), and Compass Listeria were used to enumerate the three pathogens, followed by incubation at 37 °C for the first two methods and 30 °C for the last method. After 24 to 48 h of incubation, typical colonies were enumerated. LAB were enumerated in MRS, *Enterobacteriaceae* in Violet Red Bile Glucose agar (VRBG), and Pseudomonas in Caphaloridine Fucidin Cetrimide agar (CFC). All culture media were from Biokar (Allonne, France). The results are expressed as log cfu/g, obtained by the application of the following expression:

(1) $$\log_{10}\left[\frac{cfu}{\left[\left(\frac{m}{V+m}\right)\cdot \;vi\cdot \left(\frac{1}{df}\right)\right]}\right]$$

*m*—weight of the sample (5 g). *v*—volume used in the initial dilution (20 mL). *vi*—volume (mL) used in the petri dish (0.5 low counts, 0.1 normal). *df*—dilution factor (in undiluted samples was 1).

For data analysis purposes, when the microorganism count was below the detection limit (1 log cfu/g), the count was considered to be zero. When countable colonies were present but below the recommended countable range, the counts were considered an estimate for data analysis purposes.

### 2.6. pH and Activity of Water

The pH was measured following homogenization of 10 g samples with 100 mL of deionized water in a lab mixer for 30 s (model MicropH 2002, Crison, Barcelona, Spain). Water activity was measured with a Hygroscope DT apparatus with a WA40 probe (Bassersdorf, Switzerland).

### 2.7. Data Analysis

The normality of the variable was tested using the Kolmogorof-Smirnov test. The distribution of the variable for each group defined by the effect level was non-normal. Comparisons between samples made with or without NOx was made through the Mann-Whitney test. For variables with three levels (initial contamination: LC. NC, LC + BAL; Wine: control, wine, AWS), the comparisons of the bacterial counts were made using the Kruskal-Wallis test. The differences (*p* < 0.05) between different formulations were located by the Dunn test with the Bonferroni correction (XLStat, Addinsoft). As the evolution of the pathogens is presented in graphs based on mean values, the tables presenting the statistical comparisons also present means and the corresponding standard deviation. Data analysis from part one of the experiment was performed with four repetitions (2 bags * 2 samples from each); part two of the experiment was performed with three repetitions, corresponding to three sausages per formulation and sampling time.

## 3. Results

### 3.1. Naturally Fermented Sausage Batter

**Figure 2:**
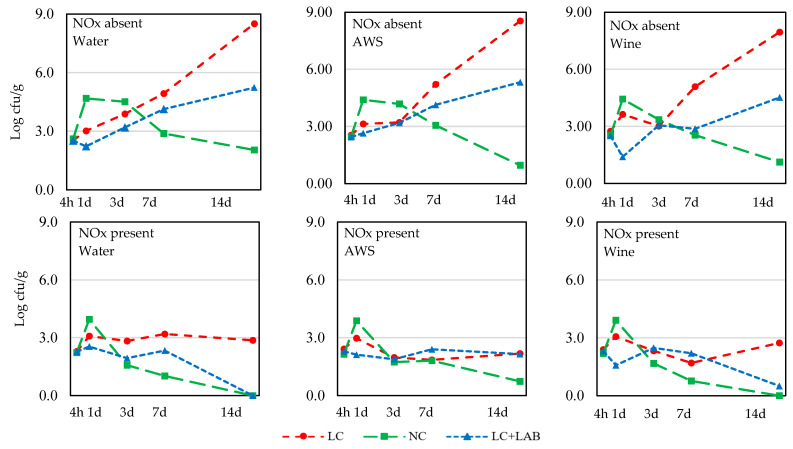
The behavior of *Salmonella* spp. during the incubation of the *chouriço* batter made from low contaminated meat (**LC**), normal contaminated meat (**NC**), and low contaminated meat with LAB added (**LC** + **LAB**), with or without nitrite plus nitrate (**NOx**) and water, artificial wine solution (**AWS**), or wine (*n* = 4).

**Figure 3:**
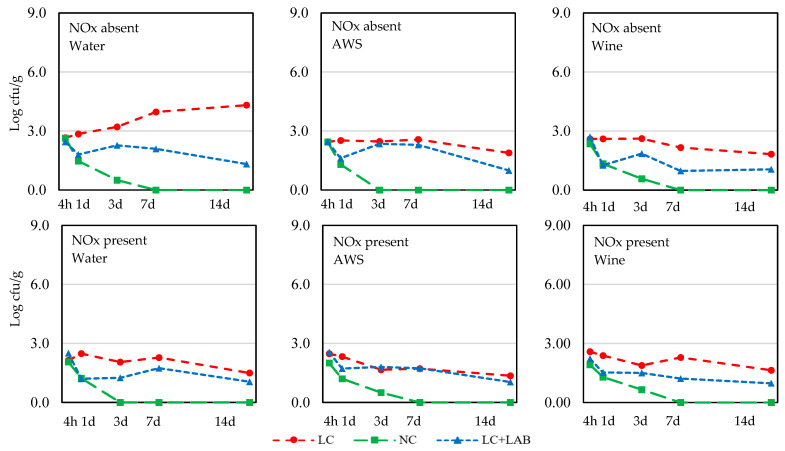
Behavior of *S. aureus* during incubation of *chouriço* batter made from low contaminated meat (**LC**), normal contaminated meat (**NC**), and low contaminated meat with LAB added (**LC** + **LAB**), with or without nitrite plus nitrate (**NOx**) and water, artificial wine solution (**AWS**), or wine (*n* = 4).

**Figure 4:**
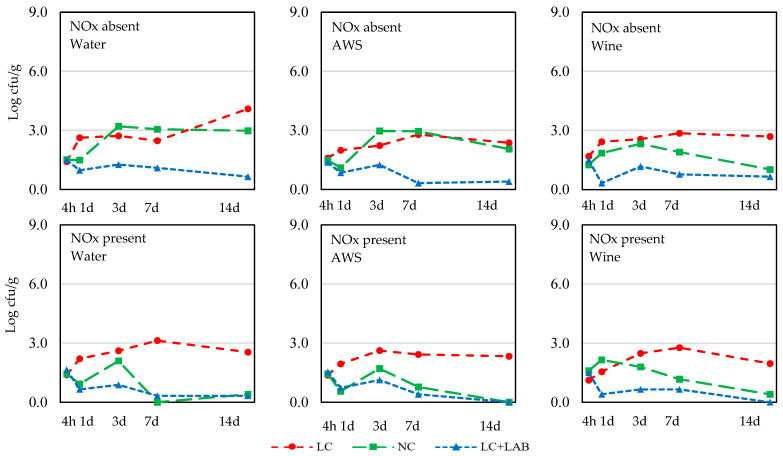
Behavior of *L. monocytogenes* during incubation of *chouriço* batter made from low contaminated meat (**LC**), normal contaminated meat (**NC**), and low contaminated meat with LAB added (**LC**+**LAB**), with or without nitrite plus nitrate (NOx) and water, artificial wine solution (**AWS**), or wine (*n* = 4).

The evolution of *Salmonella*, *S. aureus*, and *L. monocytogenes* in the batter is presented in Figure 2, Figure 3 and Figure 4, respectively. The total number of combinations of effects/levels was 18 (2 NOx * 3 wine *3 contamination levels) for each sampling time and each microorganism and the individual results are presented only in the figures. The results of the statistical comparisons are presented in Table 2.

Four hours after the batter’s preparation, the pathogens were enumerated to check the inoculation level: *Salmonella*, 2.86 ± 1.74 log cfu/g; *S. aureus*, 1.63 ± 1.13; and *L. monocytogenes*, 1.57 ± 1.08 log cfu/g. It was observed that the recently prepared batter (4 h) with NOx presented slightly lower counts of *Salmonella* and *S. aureus* (*p* < 0.05) than the batter samples without NOx (Table 2, Figure 2 and Figure 3).

The behavior of *Salmonella* during the experiment was influenced (*p* < 0.05) by the presence of NOx and by the contamination of the meat (Table 2). In the absence of NOx, particularly in LC meat, the multiplication of *Salmonella* was faster, reaching levels close to 8 log cfu/g (Figure 2). Batter prepared with LC + LAB also had considerable growth of *Salmonella* spp., which was still more controlled than in the absence of LAB. When the batter was prepared with NC meat, there was an initial growth of *Salmonella*, but after 3 days of incubation, *Salmonella* loads dropped, presenting only residual counts after 14 days of incubation. No significant difference was detected for the use of wine or AWS.

*S. aureus* showed a behavior similar to *Salmonella*, but with smaller increases in the population (Figure 3). The initial contamination level of meat clearly affected the behavior of this pathogen as the higher counts were mostly observed in the batter prepared with LC meat, followed by LC + LAB. Normal contamination meat resulted in lower survival of *S. aureus*; after 7 or 14 days of incubation, *S. aureus* was below the detection limit. The inhibitory effect of NOx was statistically significant after 4 h and 3 days of drying (Table 2), with higher inhibition in samples using the additive.

*L. monocytogenes* presented higher growth when LC meat was used in the absence of NOx (Figure 4). The effect of the contamination level was highly significant from day 1 to the end of the experiment (Table 2). When the batter was prepared without NOx, the use of a LAB starter culture contributed to the inhibition of the pathogen. This trend was also observed when NOx was used, but in this case, batters prepared with NC meat also presented low counts of *L. monocytogenes*. The effect of NOx was statistically significant from 7 days of incubation and resulted in lower counts of *L. monocytogenes*. That tendency (*p* = 0.059) was observed since the third day.

**Figure 5:**
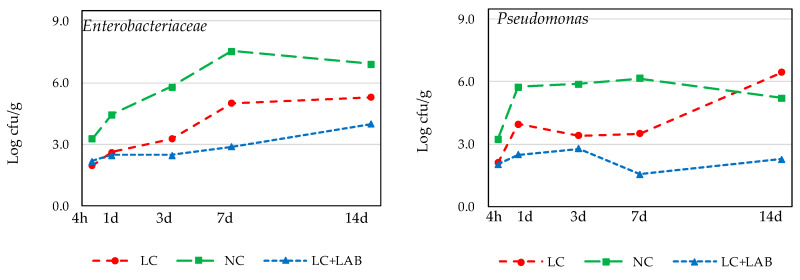
Counts of *Enterobacteriaceae* and Pseudomonas during incubation of *chouriço* batter made from low contaminated meat (**LC**), normal contaminated meat (**NC**), and low contaminated meat with LAB.

**Figure 6:**
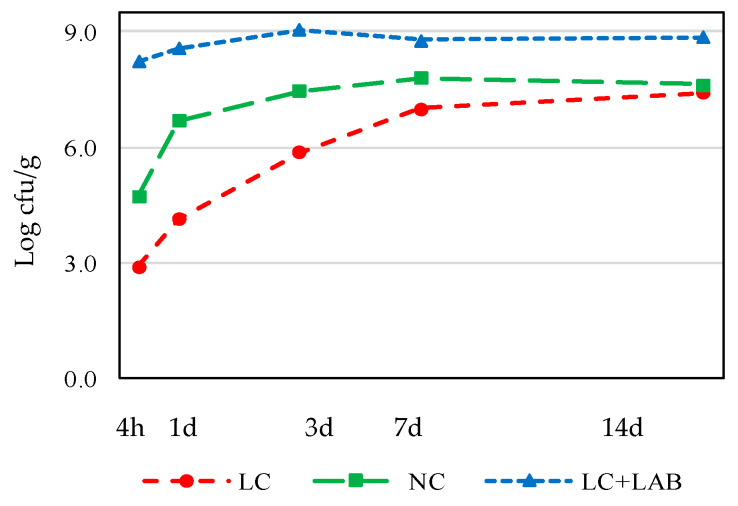
Counts of LAB during incubation of *chouriço* batter made from low contaminated meat (**LC**), normal contaminated meat (**NC**), and low contaminated meat with LAB added (**LC** + **LAB**). Combined results of samples with or without nitrite plus nitrate and water, artificial wine solution (AWS), or wine (*n* = 24).

The approach used in this part of the experiment has the limitation of challenging the pathogens in the batter without the effect of drying. However, these results allow us to understand each factor’s effect, simulating the first stages of naturally fermented sausage processing. The baseline of natural contamination of meat was around 3.27 ± 0.34 log cfu/g *Enterobacteriaceae*, 3.24 ± 0.33 log cfu/g *Pseudomonas*, and 4.75 ± 0.27 log cfu/g LAB in NC meat. The procedure used to reduce to contamination resulted in a reduction of nearly 1.2 log cfu/g for the gram-negative bacteria and 1.8 log cfu/g for the LAB. These reductions were all significant (*p* < 0.05). The overgrowth of gram-negative spoilage microorganisms (Figure 5) should be taken into consideration when analyzing these results as these organisms are highly uncommon in fermented meat products since they die when the product dries [29]. The LAB population, both in the LC + LAB or LC and NC samples, is characteristic of these products (Figure 6). At the beginning of the experiment, the difference between LC + LAB (8.23 ± 0.38 log cfu/g) and LC (2.92 ± 0.32 log cfu/g) was around 5.3 log cfu/g, which is slightly lower than the previewed inoculation of 6 log cfu/g. The difference between the previewed and observed inoculation level indicates that the *L. sakei* population suffered some lethality when introduced in the meat batters, which is common when preparing fermented sausage batters [30]. The initial count of LAB in NC was 4.75 ± 0.27 log cfu/g. As the experiment proceeded, the differences between LC, NC, and LC + BAL became narrower, to nearly 2 log cfu/g at 14 days.

It is not our intention to advocate the use of highly contaminated meat in the manufacture of naturally fermented sausages. However, one should consider not only meat with low initial microbiota due to high hygiene standards in the abattoir and carcass dressing, but also the current trend to decontaminate meat. The use of lactic and acetic acid has been considered in EU to decontaminate pig carcasses [31]. Other strategies have been proposed to decontaminate carcasses and meat based on chemical or physical treatments [32,33,34]. Considering that meat is a sensitive microbial ecosystem, these practices might result in overgrowth of pathogens that are more resistant to the treatment and the eventual emergence of pathogens that are usually of low concern [31]. The present study’s results confirm that the higher the competition in the batter, the more difficult it is for a pathogen to grow or survive, particularly if no other hurdles are introduced in the process [7,35]. This situation is of particular concern with *Salmonella*, which is an excellent competitor [36]. *S. aureus’* ability to compete has been described as limited [37], which is represented in the present results, as *S. aureus* was largely influenced by the initial contamination of meat. *L. monocytogenes* has been suggested to be a good competitor, particularly at refrigeration temperatures [38]. The present experiment was performed at 15 °C, which favors the growth of other bacteria, namely LAB. The counts of *L. monocytogenes* were maintained, in the worst scenario, at levels similar to the initial contamination, with the exception of the batter prepared without NOx and with no wine or AWS. The use of *L. sakei* as a starter culture has been confirmed to be an interesting strategy to control the multiplication of pathogens, particularly when low contaminated meat is used. Several authors have demonstrated that *L. monocytogenes* is inhibited more rapidly when LAB starter cultures are added to the manufacturing of dry-fermented sausage, among other factors, due to bacteriocins’ production, which are active against this pathogen [30]. The adaptation of LAB naturally present in the raw materials and the excellent adaptation of LAB to the sausage ecosystem results in the growth and dominance of the sausage microbiota in a few days [39]. Besides the environmental competitive advantage of LAB, the ability to eliminate competitors through bacteriocins also contributes to the interest of using LAB in starter cultures. The LAB bacteriocins are particularly active against gram-positive bacteria, which might justify the behavior of *S. aureus* and *L. monocytogenes* in the present work when *L. sakei* was used. The effect on gram-negative bacteria is variable since the outer lipopolysaccharide layer of gram-negative bacteria exerts a protective effect against the action of bacteriocins [30]. Considering the current trend in the meat industry to reduce the use of NOx [40], the results of the present experiment are worrying as the presence of NOx results in a better inhibition of the challenging pathogens. NOx is used mainly to control *Cl. botulinum* [10]. In accordance with our results, the inhibition of *Salmonella*, *S. aureus*, and *L. monocytogenes* was also demonstrated [21,41,42,43]. The broad antimicrobial effect of nitrite is due to the action of nitric oxide on Fe-S enzyme complexes, which are vital for cellular functions in several bacteria [11]. Several strategies have been proposed to replace nitrite based on vegetable extracts with high content in nitrate, based on controlled fermentation with protective microorganisms, or other innovative preservation methods [40]. The efficacy of hurdle technology has also been revisited to justify the control of *Clostridium* and other pathogens in nitrite-free, dry-cured, or dry fermented sausages [10,12,43,44].

The results observed in this part of the study cannot be compared to the pathogens’ behavior in the industry since the drying and smoking hurdles were missing. Particularly, drying has a high impact on pathogen control. The effect of the wine, or AWS, was rare or indirect. The results of the present study showed that the inhibition obtained with wine is similar to that obtained with AWS, suggesting that organic acids and ethanol are the main compounds that contribute to the inhibitory effect of wine since the AWS did not have phenolic compounds, which are reputed for their antimicrobial activity.

### 3.2. Part 2. Naturally Fermented Sausage—Chouriço

**Figure 7:**
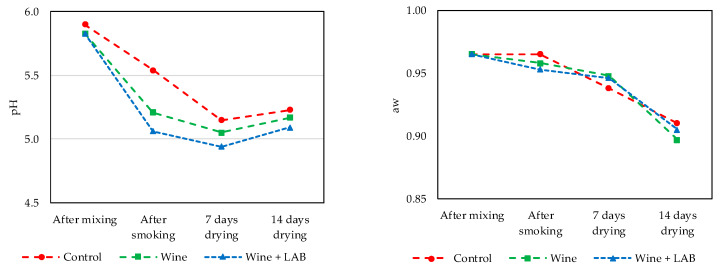
pH values (**left**) and water activity (**right**) measured in *chouriço* during processing (*n* = 3).

The *chouriço* prepared with different formulations presented a level of pathogen inoculation (4 h) between 2 and 2.5 log cfu/g (Table 3). At the end of smoking (1 day), the abundance of the three pathogens in the control samples increased slightly, while when wine was used, the abundance decreased by approximately 1 log cfu/g. The combined effect of wine and LAB resulted in counts below the detection limit for the three repetitions immediately after smoking. On the seventh day of drying, a_w_ was already below 0.95 (Figure 7), resulting in lower counts of *Salmonella* and *S. aureus*, similar (*p* > 0.05) to those found in the three types of *chouriço* tested. Lactic acid bacteria counts, 4 h after preparation, were different between non-inoculated and inoculated samples by approximately 4 log cfu/g. These differences became narrower to approximately 0.5 log cfu/g after 7 days of drying and almost disappeared at 14 days of drying. The normal spoilage microbiota of the meat was initially present in low counts and progressively disappeared. At 7 days of drying, *Enterobacteriaceae* and *Pseudomonas* were below the detection limit.

The evolution of pH (Figure 7) is characteristic of fermented sausage [30]. The reduction in pH was more evident in sausages made with wine with a low pH, and vestigial amounts of sugars also contributed to lactic fermentation [12]. *Chouriço* technology is based on natural fermentation. It is not common to add fermentable sugar to the batter, which is why the pH is never very low. The final product had a pH slightly higher than 5, which was not sufficiently reduced to be considered a stability parameter.

The results demonstrate the importance of correctly drying fermented sausages, which is the main factor responsible for its safety. The effect of wine, as well as the combination of wine with LAB, on reducing the time necessary to eliminate the pathogens of concern must be stressed as it allows the producer to eventually deem the product finished, even with a slightly higher a_w_ than usual, improving juiciness and yield.

## 4. Conclusions

The results of the present study performed with naturally fermented sausage batter revealed that the reduced contamination in raw meat might be a favorable factor for the multiplication of pathogens. The inhibitory effect of NOx on *Salmonella*, *S. aureus*, and *L. monocytogenes* was confirmed. This additive showed increased importance for achieving inhibition as the competition in meat was lower. Thus, any attempt to reduce or eliminate NOx from dry-cured sausages should consider the use of LAB starter cultures to ensure an unfavorable competition environment for the pathogens that will eventually be present. When the naturally fermented sausages were dried, a_w_ reduction resulted in a strong inhibition of the tested pathogens. That inhibition was more effective when red wine was used or when used in combination with the LAB starter culture.

## Figures and Tables

**Table 1:** Strains used in the experiment.

*Salmonella*	*S. aureus*	*L. monocytogenes*
CECT 4155	ATCC 25923	NCTC 7973
MPI-B-S07 ^1^ (*Chouriço* batter)	EDS-B-LM05 ^1^ (*Chouriço* batter)	EDS-ChM-SA01 ^1^ (*Chouriço* at the middle of the drying process)
EDS-E-S26 ^1^ (Environment of meat products preparation)	MPI-E-LM18 ^1^ (Environment of meat products preparation)	EDS-E-SA07 ^1^ (Environment of meat products preparation)

^1^ Strains isolated from meat products or the environment of its production are from our laboratory collection.

**Table 2:** Comparisons of the counts of the three pathogens in *chouriço* batter made with or without curing salts (NOx), with wine or artificial wine solution (AWS) or water, using low contaminated meat (LC), normal contaminated meat (NC), and low contaminated meat inoculated with *L. sakei* (LC + LAB). The results are expressed as the mean ± standard deviation of log cfu/g.

Phase	NOx			Wine				Contamination			
	Absent (*n* = 36)	Present (*n* = 36)	*p*	Water (*n* = 24)	AWS (*n* = 24)	Wine (*n* = 24)	*p*	LC (*n* = 24)	NC (*n* = 24)	LC + LAB (*n* = 24)	*p*
***Salmonella***											
4 h	2.54 ± 0.23	2.28 ± 0.37	0.001	2.41 ± 0.41	2.38 ± 0.25	2.44 ± 0.32	ns	2.48 ± 0.43	2.36 ± 0.30	2.39 ± 0.24	ns
1 d	3.28 ± 1.19	3.01 ± 0.96	ns	3.25 ± 0.88	3.19 ± 0.95	3.00 ± 1.38	ns	3.15 ± 0.30 b	4.21 ± 0.41 c	2.08 ± 1.01 a	<0.001
3 d	3.51 ± 1.16	2.05 ± 1.27	<0.001	2.99 ± 1.41	2.70 ± 1.41	2.65 ± 1.46	ns	2.87 ± 1.89	2.84 ± 1.97	2.62 ± 0.93	ns
7 d	3.87 ± 1.31	1.92 ± 1.32	<0.001	3.08 ± 1.65	3.08 ± 1.59	2.52 ± 1.67	ns	3.67 ± 1.67 b	2.01 ± 1.80 a	3.01 ± 0.89 ab	0.007
14 d	4.91 ± 3.08	1.24 ± 1.40	<0.001	3.11 ± 3.21	3.31 ± 3.01	2.80 ± 2.30	ns	5.46 ± 3.04 b	0.80 ± 1.49 a	2.95 ± 2.27 a	<0.001
***S. aureus***											
4 h	2.53 ± 0.27	2.27 ± 0.53	0.010	2.41 ± 0.60	2.39 ± 0.34	2.39 ± 0.34	ns	2.48 ± 0.59	2.23 ± 0.30	2.48 ± 0.32	ns
1 d	1.86 ± 0.86	1.71 ± 0.75	ns	1.84 ± 0.90	1.78 ± 0.71	1.73 ± 0.82	ns	2.58 ± 0.29 b	1.30 ± 0.83 a	1.52 ± 0.58 a	<0.001
3 d	1.76 ± 1.21	1.25 ± 0.95	0.017	1.55 ± 1.24	1.46 ± 1.05	1.51 ± 1.07	ns	2.31 ± 0.64 b	0.37 ± 0.85 a	1.84 ± 0.72 b	<0.001
7 d	1.56 ± 1.39	1.22 ± 1.05	ns	1.68 ± 1.43	1.38 ± 1.19	1.11 ± 1.03	ns	2.49 ± 0.95 c	<DLa	1.67 ± 0.66 b	<0.001
14 d	1.27 ± 1.65	0.84 ± 0.90	ns	1.36 ± 1.54	0.88 ± 1.27	0.92 ± 1.18	ns	2.09 ± 1.60 b	<DLa	1.07 ± 0.82 a	<0.001
***L. monocytogenes***											
4 h	1.48 ± 0.39	1.44 ± 0.56	ns	1.48 ± 0.56	1.47 ± 0.40	1.44 ± 0.37	ns	1.43 ± 0.68	1.45 ± 0.29	1.51 ± 0.22	ns
1 d	1.51 ± 0.99	1.23 ± 0.99	ns	1.48 ± 0.99	1.19 ± 1.00	1.45 ± 1.01	ns	2.12 ± 0.59 c	1.34 ± 1.02 b	0.65 ± 0.74 a	<0.001
3 d	2.19 ± 0.95	1.77 ± 0.88	0.059	2.13 ± 1.04	1.98 ± 0.88	1.83 ± 0.89	ns	2.54 ± 0.44 b	2.35 ± 0.71 b	1.06 ± 0.79 a	<0.001
7 d	2.02 ± 1.27	1.29 ± 1.24	0.017	1.68 ± 1.48	1.61 ± 1.28	1.69 ± 1.16	ns	2.74 ± 0.73 c	1.64 ± 1.31 b	0.59 ± 0.73 a	<0.001
14 d	1.88 ± 1.40	0.88 ± 1.67	0.003	1.83 ± 1.57	1.19 ± 1.25	1.12 ± 1.21	ns	2.66 ± 0.87 b	1.14 ± 1.34 a	0.34 ± 0.60 a	<0.001

a, b, c, Means in the same line under the same heading are different (*p* < 0.05). ns, not significant.

**Table 3:** Counts of the three pathogens in the present study and LAB in *chouriço* made without wine (control), supplemented with 7.5% red wine (wine) and with the additional inoculation of 6 log cfu/g *L. sakei* (Wine + LAB). The results are expressed as the mean ± standard deviation of log cfu/g.

Microorganism Phase of Processing	Control (*n* = 3)			Wine (*n* = 3)			Wine + LAB (*n* = 3)			*p*
***Salmonella***										
After mixing (4 h)	2.50	±	0.35	2.32	±	0.28	2.55	±	0.13	ns
After smoking (1 day)	3.04	±	0.19	1.01	±	1.76	<LD			0.078
7 days drying	1.87	±	1.63	0.82	±	1.43	<LD			ns
14 days drying	<LD			<LD			<LD			-
***S. aureus***										
After mixing (4 h)	2.20	±	0.17	2.16	±	0.28	2.47	±	0.40	ns
After smoking (1 day)	2.59	±	0.26	1.43	±	1.25	<LD			0.054
7 days drying	0.67	±	1.15	1.33	±	1.15	<LD			ns
14 days drying	<LD			<LD			<LD			-
***L. monocytogenes***										
After mixing (4 h)	2.20	±	0.17	2.46	±	0.45	2.26	±	0.24	ns
After smoking (1 day)	3.10	±	0.44 b	1.59	±	1.38 ab	<LD	a		0.034
7 days drying	2.65	±	0.16 b	1.43	±	1.25 ab	<LD	a		0.034
14 days drying	2.16	±	0.28	0.93	±	1.60	<LD			ns
**LAB**										
After mixing (4 h)	2.22	±	0.07 ab	2.05	±	0.10 a	6.14	±	0.53 b	0.027
After smoking (1 day)	5.67	±	0.00 ab	4.83	±	0.01 a	7.73	±	0.03 b	0.024
7 days drying	6.55	±	0.01 a	7.39	±	0.01 b	7.10	±	0.04 ab	0.027
14 days drying	7.10	±	0.04 ab	6.87	±	0.04 a	7.48	±	0.1 b	0.044
***Enterobacteriaceae***										
After mixing (4 h)	2.28	±	0.11 a	1.22	±	0.24 ab	0.73	±	0.63 b	0.048
After smoking (1 day)	1.77	±	0.11 a	0.57	±	0.51 ab	<LD	b		0.034
7 days drying	1.45	±	0.26	<LD			<LD			0.022 ^Ψ^
14 days drying	<LD			<LD			<LD			-
***Pseudomonas***										
After mixing (4 h)	2.74	±	0.13 a	2.40	±	0.20 ab	2.19	±	0.20 b	0.044
After smoking (1 day)	2.72	±	0.26 a	0.57	±	0.98 ab	<LD	b		0.035
7 days drying	1.33	±	0.15	<LD			<LD			ns
14 days drying	<LD			<LD			<LD			-

a, b, c, means in the same line with different letters are different (*p* < 0.05); ns, not significant; ^Ψ^ although the *p*-value is significant, the individual differences were not significant.
